# Bio-inspired artificial synapse for neuromorphic computing based on NiO nanoparticle thin film

**DOI:** 10.1038/s41598-023-33752-5

**Published:** 2023-05-09

**Authors:** Keval Hadiyal, Ramakrishnan Ganesan, A. Rastogi, R. Thamankar

**Affiliations:** 1 Centre for Functional Materials, Vellore Institute of Technology, Vellore, TN 632014 India; 2 Department of Physics, School of Advanced Sciences, Vellore Institute of Technology, Vellore, TN 632014 India; 3 Department of Chemistry, Birla Institute of Technology and Science (BITS), Pilani, Hyderabad Campus, Jawahar Nagar, Kapra Mandal, Medchal District, Hyderabad, Telangana 500078 India

**Keywords:** Engineering, Materials science, Nanoscience and technology, Physics

## Abstract

The unprecedented need for data processing in the modern technological era has created opportunities in neuromorphic devices and computation. This is primarily due to the extensive parallel processing done in our human brain. Data processing and logical decision-making at the same physical location are an exciting aspect of neuromorphic computation. For this, establishing reliable resistive switching devices working at room temperature with ease of fabrication is important. Here, a reliable analog resistive switching device based on Au/NiO nanoparticles/Au is discussed. The application of positive and negative voltage pulses of constant amplitude results in enhancement and reduction of synaptic current, which is consistent with potentiation and depression, respectively. The change in the conductance resulting in such a process can be fitted well with double exponential growth and decay, respectively. Consistent potentiation and depression characteristics reveal that non-ideal voltage pulses can result in a linear dependence of potentiation and depression. Long-term potentiation (LTP) and Long-term depression (LTD) characteristics have been established, which are essential for mimicking the biological synaptic applications. The NiO nanoparticle-based devices can also be used for controlled synaptic enhancement by optimizing the electric pulses, displaying typical learning-forgetting-relearning characteristics.

## Introduction

The last few decades have seen tremendous technological advancement, thanks to the capability of fabricating smaller and smaller electronic devices for various applications. The manufacturing of high-density architecture is borrowed from the ideas of Allen Turing, who proposed computation using serial instructions. The von Neuman architecture puts forward a more successful route for information storage and information processing with both storage and processing occurs are separate physical locations^1^. The von Neuman architecture not only has an information processing time but also has energy consumption limitations. Since the complementary metal oxide semiconductor technology, popularly known as CMOS technology is reaching its lowest limit, newer and superior technology is expected to become prominent in the future with better performance. It has been proved over the years that device scaling alone will not solve the problem of processing time and energy consumption. The continuous exchange of information between the memory unit and the processing unit has its limitation referred to as the von Neumann bottleneck, which ultimately will become the reason for the limit for miniaturization. With the exponential growth of data availability and the requirement of big data processing, the fundamental limitation of processing speed and energy efficiency has posed doubt on the success of von Neumann architecture for the future of information processing and computing devices.

To overcome the limitations set by the von Neuman architecture, we need better and efficient design of the electronic architecture and to do that we can get inspiration from the human brain. Here, information storage and processing occur at the same physical location, which completely removes the necessity of information passage which is time and energy-consuming^2^. At the core of brain operation, there is a pre-synaptic area (neuron) and a post-synaptic area (neuron) connected with a controlling synapse. The synapse tunes the strength of the connection between the two synaptic terminals. The information storage and processing occur completely differently in this neuron–synapse–neuron (N–S–N) combination. In addition, the working of a human brain is characterized by massive parallel connections of neurons which results in parallel computing with the highest energy efficiency^3^. In this regard, there are efforts to fabricate solid-state devices which can mimic the working principle of a fundamental unit of the human brain. Such device fabrication is intended to store memory and process the information in the same physical location, which will replicate the basic functionalities of the brain, such as learning and memory (short-term and long-term). Thus, the ‘Brain inspired’ or ’Neuromorphic’ computing could potentially revolutionize electronics and computing devices in the future^4–19^.

The fundamental unit of the human brain can be a simple neuron–synapse–neuron (N–S–N) system. The neurons transmit the signals and the synapse either amplifies or reduces the signal which is to be transmitted from one neuron to the other. It is estimated that the human brain consists of approximately 10$^{11}$ neurons and $\approx$ 10$^{15}$ synapses with the parallel interconnection of extremely high density^20–26^. Each neuron is interconnected with hundreds of other neurons using synapses. The ability of the synapses to tune the strength or amplitude of the connection between the neurons is the basic idea of learning and the memory. Interesting to note that both learning and memory happen in the synapse which is the key point in the working principle of the neuron–synapse–neuron system. That means one needs to find an equivalent electronic device that can modulate its conduction state and also have memory functionalities suitable for mimicking the N–S–N system. In the last decade, extensive research has been done to emulate the biological neurons and ‘artificial’ synapse and understand the basic memory functionalities^13–19^. One of the simplest device structure which can show the memory and processing at the same location is memristor^27^. In its simple geometry, a memristor is a two-terminal device where an insulating material is sandwiched between the two metal electrodes. One can emulate the fundamental operation of neuron–synapse–neuron system using this simple geometry containing an insulating material sandwiched between two electrodes. The metal electrodes will send and receive the information and sandwiched layer will store and process the information. The special feature of the insulating layer is that it can retain various resistance states depending on the training procedure using voltage or current, making sure that the information is stored at different levels. The ability to retain different conductance levels by memristor precisely compares well with the N–S–N operation with various synaptic weights in biological synapses. Many efforts have been put into fabricating a replica of the N–S–N-like device in the laboratory. There are mainly two types of devices that can emulate the N–S–N functions. (i) the metal–oxide–metal (M–I–M) combination in the form of vertical or lateral device geometry or, simply, a two-terminal device. Here, the conductance state of the device is mapped with applied voltage. (ii) a transistor geometry or a three-terminal device. In this case, the third terminal acts as a control gate. With this design, the channel conductance can be controlled/tuned much more efficiently. It has been established now that the conductance states change abruptly (digital switching) or gradually (analog switching) depending on the switching material considered.

Resistive switching phenomena has been studied in a variety of materials such as binary oxides like HfO$_{2}$, CeO$_{2}$, TaO$_{2}$, TiO$_{2}$, CrO$_{2}$, SiO$_{2}$, Ga$_{2}$O$_{3}$ and ZnO, NbO$_{2}$ and perovskite oxides such as SrTiO$_{3}$ and spinel oxides such as NiFe$_{2}$O$_{4}$, CoFe$_{2}$O$_{4}$, Zn$_{2}$SnO$_{4}$^23,28,29^, Chalcogenide based thin film devices, carbon-based materials, and two-dimensional materials such as MoS$_{2}$, h-BN and their heterostructures^5,25,30–38^. During the operation of a memristor, electroforming step is applied at the beginning and then the resistance state is uncontrollably switched between the high-resistance state (HRS) and low-resistance state (LRS). There are mainly two switching categories. (i) unipolar switching, where the magnitude of the electric field determines the resistance state rather than the polarity and (ii) Bipolar switching depends on the polarity of the external electric field.

The binary oxide NiO-based thin film devices have been studied extensively for resistance switching. A detailed model for the conduction mechanism in the RRAM cells using NiO has been extensively discussed^39–41^. There are various conduction mechanisms possible in oxide-based memristor devices. Majority of results consider the trap-assisted tunneling (TAT model), space-charge-limited conduction (SCLC), and joule heating as the major conduction mechanisms. Even though the type of conduction mechanisms are still a topic of discussion, the formation and dissolution of conduction filament and/or charge trapping/de-trapping (TAT model) are considered two possible mechanisms of conduction due to the application of the electric field^42^. There are extensive reports on resistive switching in NiO thin films where the conduction is primarily from the oxygen ion vacancies is considered. The origin of such oxygen vacancies is attributed to the process-induced vacancies, which outnumber vacancies formed during the creation of the conduction filament. NiO grows as Ni-deficient oxide film, and it is found that the oxygen vacancies contribute electrons to the Ni-vacancies. Due to the very high electric field across the conduction filament, oxygen migration can create an oxygen vacancy. This oxygen vacancy will have a +2 charge, giving two localized electrons to the nearby Ni atoms. The two electrons will reduce the Ni$^{2+}$ to Ni$^{0}$, thereby creating a metallic conduction path representing the ON state of the device. If the device structure is such that there is a large difference in the work functions of electrodes and the switching material, then energy band bending near to the interface is expected. Due to the band bending across the interface, trap-assisted tunneling dominates the conduction process. In our case, we have selected the Au electrodes whose work function matches very well with the NiO nanoparticle films^43,44^. (The work function of NiO nanoparticles and Au is 5.37 eV and 5.38 eV, respectively). This implies our devices should show negligible band bending at the interface of NiO/Au, which is associated with negligible trapping and de-trapping of charge carriers at the interface. In the case of NiO thin films, it is found that the charge compensation of V$_{Ni}$ is the main driving mechanism for the p-type conductivity^45^. The solution processed NiO$_{x}$ nanoparticles have been successfully used for efficient hole transport layer in organic optoelectronic devices^46^. There are few reports on NiO thin film-based resistive switching devices and few attempts in using NiO thin film devices for synaptic applications^39–41,47^. A modified Ebbinghaus forgetting curve has been established for NiO thin film memristor with conductance decreasing with time^48^. Recently, habituation and sensitization properties of NiO-based devices have been studied under the exposure of 5$\%$ H$_{2}$ exposure^40^. To our understanding, there are no reports on studies involving resistive switching and the possible usage of NiO nanoparticle thin films for artificial synaptical applications.

## Results

**Figure 1:**
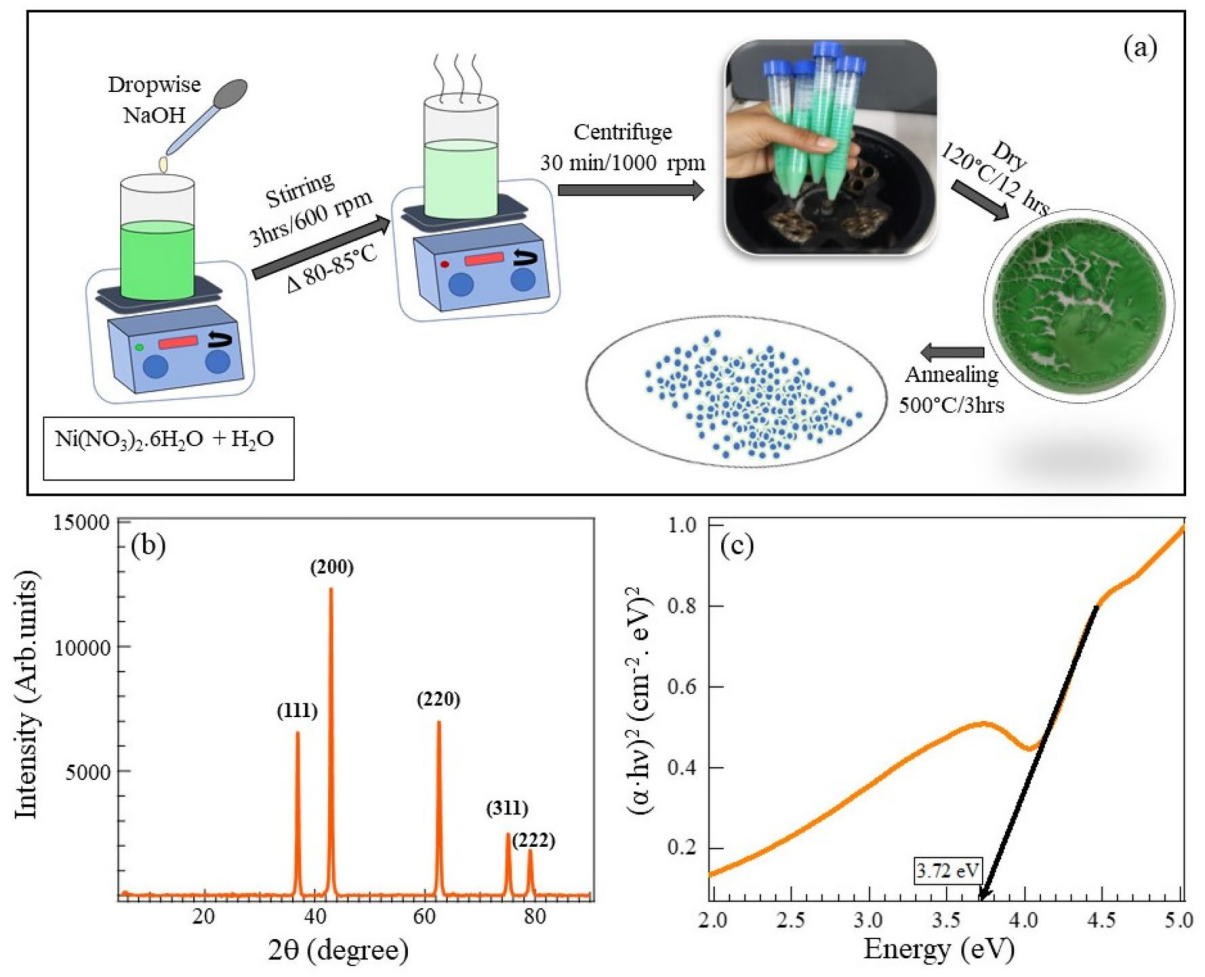
(**a**) Schematic diagram of the synthesis procedure of NiO nanoparticles. The details are given in the text. (**b**) X-ray diffraction spectra of cleaned NiO nanoparticles. The high-intensity peaks have been identified at 2$\theta$ = 36.9$^{\circ }$, 42.97$^{\circ }$ are indexed to (111) and (200) crystallographic planes. (**c**) The UV–vis spectra converted into a Tauc plot indicate an optical energy gap of 3.72 eV.

**Figure 2:**
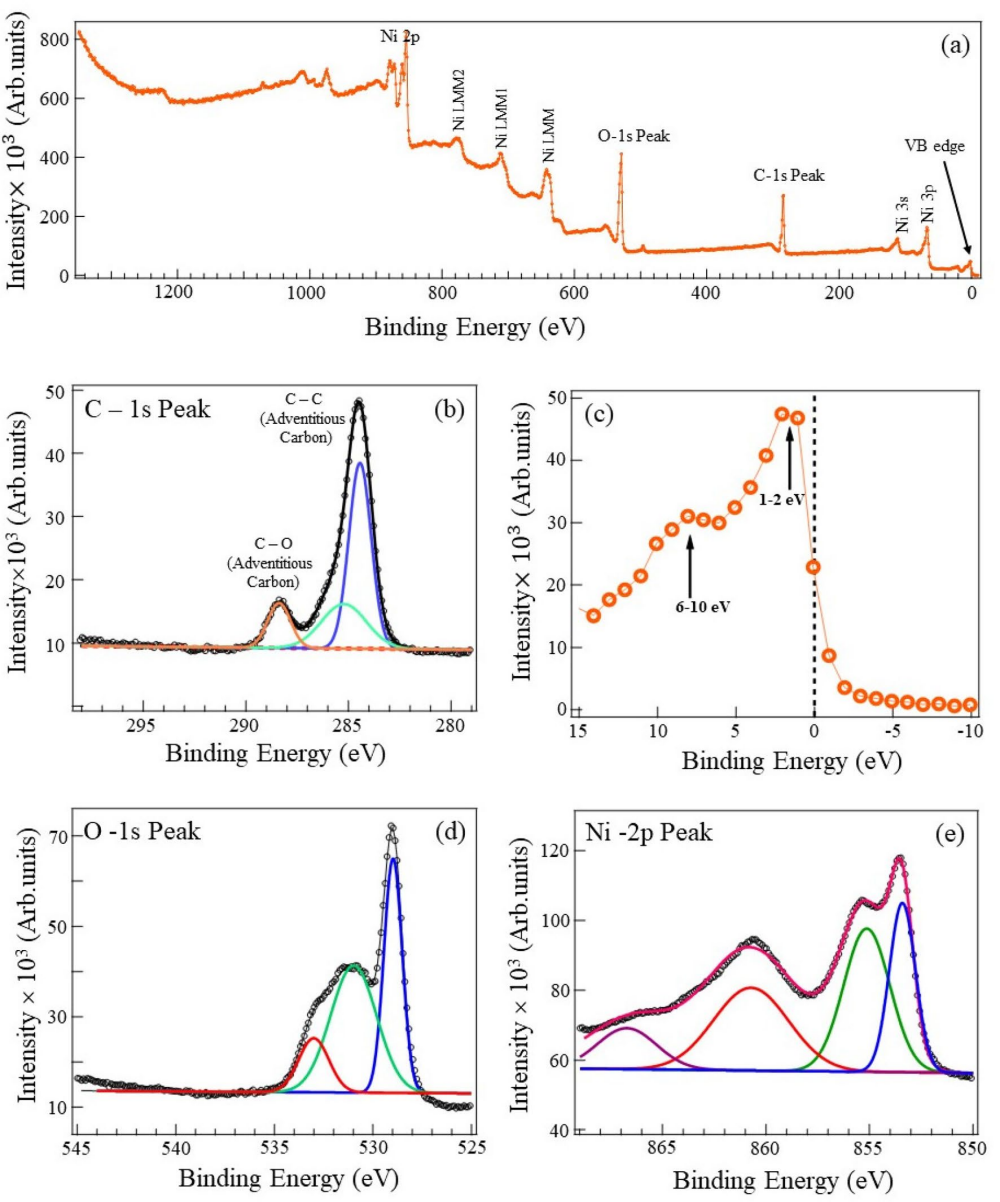
Element-specific x-ray photoelectron spectroscopy of NiO nanoparticle film. (**a**) Survey scan of NiO. A clear valence band edge can be seen close to Fermi energy. Further, on the left, spectral features corresponding to the Carbon peak and the Oxygen peaks are denoted. (**b**) high resolution scan close to the binding energy of Carbon. This arises from the handling of the samples during the XPS measurements, referred to as “adventitious carbon peaks”. (**c**) A high resolution XPS scan of the valence band edge shows the Ni-3d orbital states within − 1.0 to − 2.0 eV. The O-2p states significantly contribute to the energy range between 6 eV to 10 eV. (**d**) High resolution scan close to the binding energy of Oxygen peaks. The peaks at 529 eV, 531 eV, and 533 eV are attributed to O$^{2-}$, O$^{1-}$, and the chemically adsorbed oxygen ions. (**e**) High resolution XPS scans of Ni binding energy. Peaks at 853 eV, 855 eV, and broad shoulder at 861 eV are due to 2P$_{3/2}$.

The purity of the as-synthesized NiO nanoparticles was checked using powder X-ray diffraction. Figure 1b shows a typical x-ray diffraction spectra of as-prepared NiO nanoparticles. The peak positions at 2$\theta$ = 36.9$^{\circ }$, 42.97$^{\circ }$, 62.57$^{\circ }$, 75.17$^{\circ }$ and 79.12$^{\circ }$ are indexed as (1 1 1), (2 0 0), (2 2 0), (3 1 1), and (2 2 2) crystallographic places of bulk NiO, respectively. The diffraction peaks and relative intensities match very well with the face-centered cubic (FCC) phase of NiO (JCPDS, No. 04-0835). It is important to note that the diffraction pattern confirms the pure phase of NiO without any impurity phases. The average crystallite size of the nanoparticles was calculated using Debye–Scherrer’s equation $\textit{D}$ = $\frac{K \lambda }{B \cos \theta }$ , where *D* represents the crystallite size, K = 0.94 is the Scherrer’s constant of the (hkl) planes of the crystallite; $\lambda$ is the wavelength of the X-rays. The average crystallite size is $\sim$23 nm, as shown in the table(S2). To estimate the optical energy gap, we performed the UV-vis spectroscopy and converted it into a Tauc plot, as shown in Fig. 1c. The extrapolation of the band to the energy axis shows 3.67 eV. A detailed insight into the electronic structure and composition of NiO is derived from the X-ray photoelectron spectroscopy measurements. The survey XPS spectra presented in Fig. 2a, reveal the presence of Nickle and Oxygen. Firstly, we note that there are carbon peaks at 284.52 eV and 288.34 eV as shown in Fig. 2b, respectively, which come from the C-C and C-O bonds. We attribute such carbon peaks as adventitious carbon peaks (AdC) coming from the surface contamination and the handling of the sample during the XPS measurements^49^. The high-resolution XPS spectra near Fermi energy give us details about the presence of Ni and O, and as shown in Fig. 2c, the peaks of Ni (1–2 eV) and a broad peak of O (6–10 eV). The high-resolution Ni spectra in Fig. 2e shows the primary peak at 853.54 eV of 2P$_{3/2}$ and a shoulder at 855 eV with a satellite peak at 860.6 eV. Similarly, the 2P$_{1/2}$ peaks at 872.3 eV, and 879 eV can also be identified. If one observes the high-resolution oxygen spectra in Fig. 2d, it can be fitted using three gaussian functions with binding energies(BEs) of approximately 529 eV, 531 eV, and 533 eV. We attribute these peaks to the O$^{2-}$ at 529 eV, the O$^{1-}$ ions at 531 eV, and the chemically absorbed oxygen ions at 533 eV^50^. The peak at 855 eV in the Ni 2P$_{3/2}$ spectra could be due to the presence of Ni$^{3+}$ ions, and the peak of O 1s at 531 eV could be assigned to excess oxygen in the form of NiO-OH. It is well established that the peak at 853 eV due to Ni 2P$_{3/2}$ and the lattice incorporated oxygen (1s) peak at 528 eV are coming from the Ni–O octahedral bonding in the cubic rock salt structure in NiO^51^. In the case of NiO nanoparticles, the XPS spectra indicate the presence of Ni$^{3+}$, and the oxygen ion vacancies^52,53^. This will encourage the adsorption of oxygen, trapping the conduction electrons.

### Conduction mechanism via DC voltage stress

**Figure 3:**
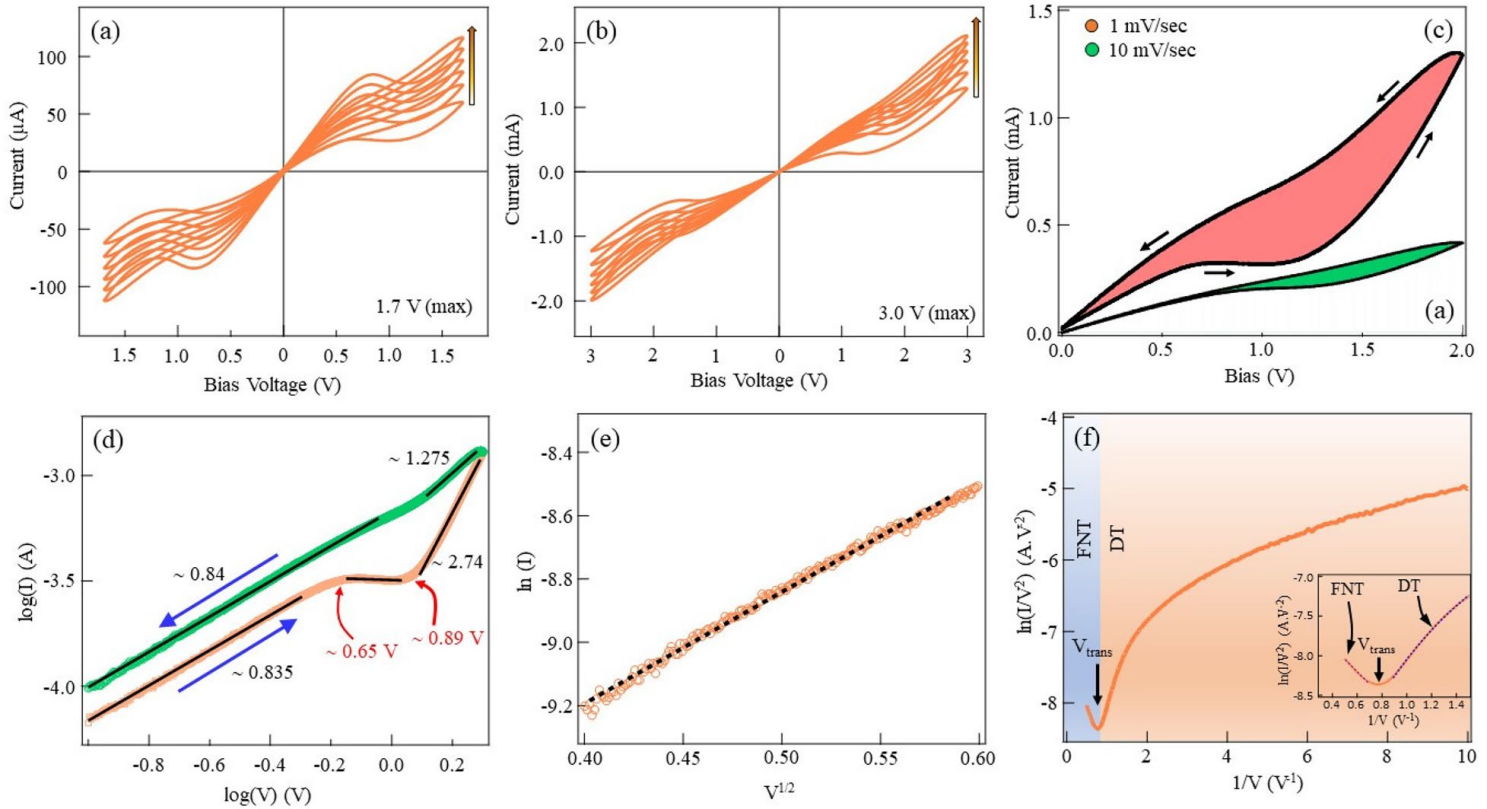
(**a**) A current–voltage measurement of the Au–NiO–Au device. Memristive characteristics with gradual switching is shown by the device. With the continuous cyclic operation, the conductivity increases in the devices. This is observed independent of the bias range selected, as shown in (**a**,**b**). (**c**) Different voltage scanning speeds result in similar I–V characteristics with gradual switching of the resistance state in the device. For slow scanning speed, larger hysteresis is observed, and for a faster scanning rate, smaller hysteresis can be seen. (**d**) The conduction mechanism via the NiO nanoparticle thin films is evaluated by plotting log I–log V. Voltage range where different slopes of I–V are indicated. Small voltage region is characterized by Ohmic conduction. For higher voltage, the slopes increase higher values indicating different conduction mechanisms. (**e**) Trap Assisted Tunneling (TAT) model was applied to the conductivity of the NiO-based device. (**f**) The high voltage conduction indicates F–N tunneling and a transition to direct tunneling at smaller voltages. The transition is indicated by the minimum at V$_{trans}$ = 1.3 V.

To understand the conduction mechanism in NiO nanoparticle films, current–voltage (dc) measurements were performed. The voltage was applied to one of the Au electrodes with the other Au electrode grounded. A typical current–voltage (I–V) curve in Fig. 3a shows analog switching without the necessity of electroforming. We performed measurements with various voltage ranges, and the devices show similar switching. The current level increased when multiple voltage cycles are applied. As shown in Fig. 3a,b, a progressive increase in the current was observed when the measurements were repeated. The increase in the current level with the voltage cycle is observed in the negative bias direction as well. since our devices are symmetric, such kind of symmetric current–voltage characteristics are expected. Our devices show typical analog switching independent of the scan range and scan speed. For example, Fig. 3c shows the scan rate-dependent current–voltage measurements with faster scanning resulting in smaller area in the current–voltage hysteresis. For clarity, current–voltage characteristics for the scan rates of 1 mV/s to 10 mV/s are shown. For the faster scan rate (10 mV/s), the current remained in the order of several $\mu$A, while for the slower scan rate (1 mV/s), the current increased to the *mA* range. The area of the hysteresis was reduced when the scan rate was increased from 1 mV/s to 10 mV/s, as shown in Fig. 3c. All the I–V characteristics obtained for different voltage ranges resulted in similar non-linear I–V curves. To understand the conduction mechanism in NiO nanoparticle film, we plotted the current–voltage characteristics in the form as shown in Fig. 3d–f. Generally, more than one mechanism must be invoked to understand the conduction in the devices. In our devices, the I–V curve (Fig. 3d) in the high-resistance state (HRS) comprises the Ohmic-like conduction (I$\propto$ V$^{0.835}$) part, which is dominated by thermally generated carriers. This is followed by a space charge limited conduction (SCLC) mechanism, which corresponds to Child’s law. Devices also show Schottky emission indicated by the linear fitting the ln(I)–V$^{0.5}$. Further, at higher bias voltages, we found a negative slope in the ln(I/V$_{2}$) - 1/V plot confirming the Fowler–Nordheim (F–N) tunneling. Extending the graph to a larger voltage range clearly shows a transition to F–N tunneling (Fig. 3f) indicated by blue shaded reagion. The transition from the direct tunneling to F–N tunneling is characterized by a minimum in the ln(I/V$_{2}$) - 1/V curve, which is normally indicated as V$_{trans}$. In our Au/NiO/Au nanoparticle film devices, this transition from direct tunneling to F–N tunneling occurs at V$_{trans}$ $\simeq$ 1.3 V shown by the minimum of the curve.

### Emulating biological synapse

**Figure 4:**
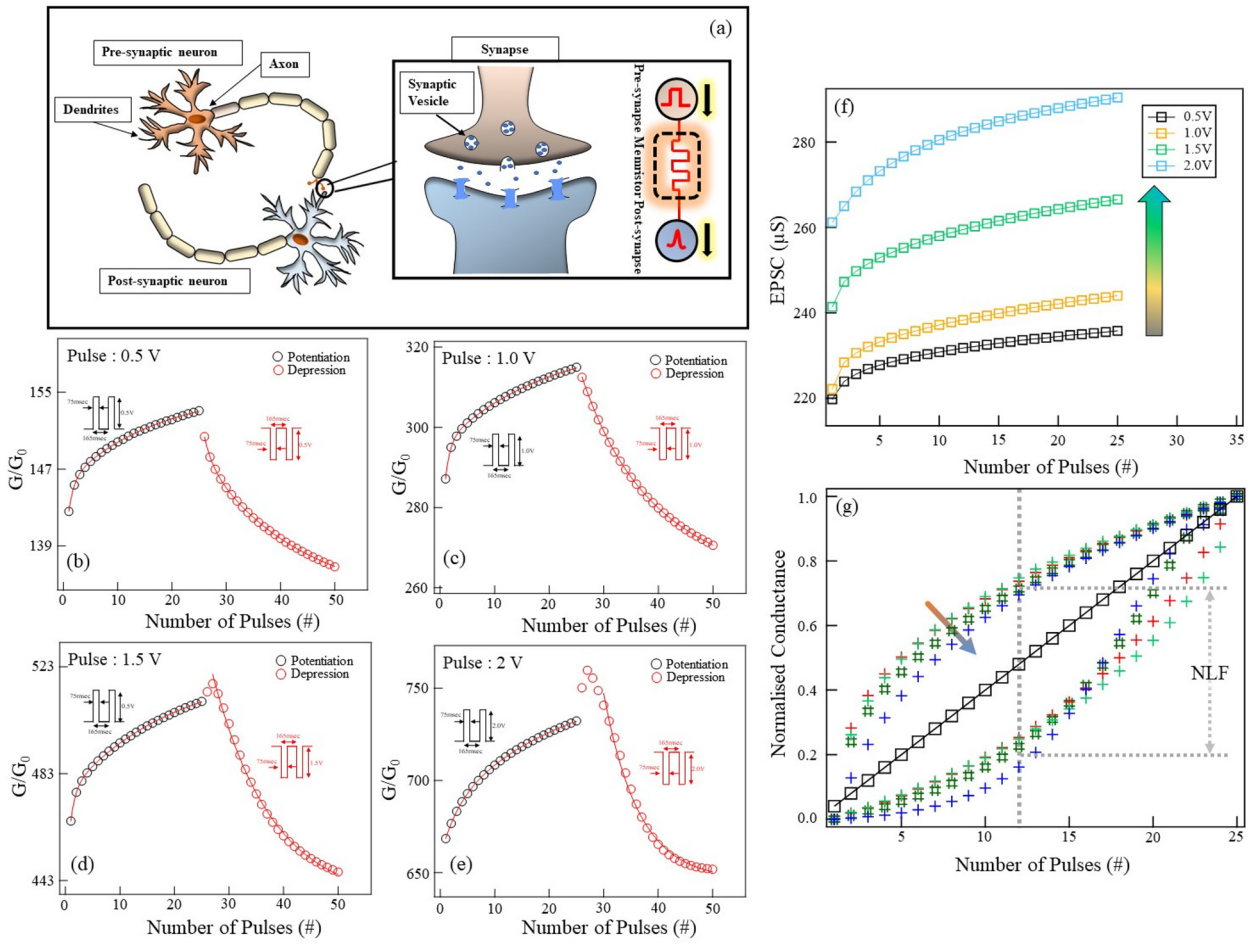
Overview of the potentiation (Learning) and Depression (forgetting) curves measured on Au/NiO/Au device. (**a**) A schematic diagram of a biological synapse. A pre-synaptic neuron is connected to a post-synaptic neuron at the synapse, which becomes a point of contact. A more detailed schematic diagram indicates the synaptic weight is modulated via molecular migration through a synapse. This happens when an action potential is applied in the pre-synaptic neuron. The pre-synaptic and post-synaptic neuron connection is mimicked in a solid state via a memristor, as shown. (**b**–**e**) are the potentiation and depression curves for various voltage pulses. In each measurement, a series of 25 pulses are applied. The sequence of voltage pulses is indicated in the inset. The continuous red curve indicates a double exponential fitting used for both potentiation and depression curves, respectively. V$_{pulse}$ = 75 ms and V$_{p-p}$ = 165 ms. (**f**) The dependence of the EPSC with the pulse voltage. A monotonic increase in the current with the magnitude of pulse voltage can be seen in the device. (**g**) The normalized conductance plot for potentiation and depression for four voltage pulses was used in the experiment. Pulse number 12 is considered for calculating nonlinearity in each potentiation and depression cycle.

As shown in Fig. 3, the Au/NiO/Au devices show a gradual resistive switching (or analog switching) behavior. The analog resistive switching completely mimics biological synapses. This means our Au/NiO/Au devices can be utilized for synaptic functionalities for neuromorphic computation. The fundamental building blocks of neuromorphic activity are the neurons and synapses. Mimicking the brain activity via neurons and synapses and their extreme parallel interconnections form the basis of neuromorphic hardware and neuromorphic computation. The neuron–synapse–neuron combination is replicated in the laboratory by employing a simple capacitor like metal-insulator-metal system. A schematic diagram of the pre-synaptic neuron to the post-synaptic neuron connected through a synapse is shown in Fig. 4a. The learning and memory functions in the brain are controlled by the weight modulation in the synapse. Such synaptic modulation can be controlled by applying systematic pre-synaptic and post-synaptic spikes. In order to determine the synaptic behavior in our device, a train of constant voltage pulses was applied on one Au electrode, and other electrode was kept at ground potential. As shown in Fig. 4b–e, we applied consecutive voltage pulse trains of different magnitudes and monitored the relative conductance (G/G$_{0}$) of the device. Here, we us G$_{0}$ = 7.75 $\times$ 10$^{-5}$ S as quantum of conductance. For positive voltage pulses, the conductance increased, while for the negative pulses, the conductance reduced. This trend can be seen for all the voltage pulses in our measurements. The continuous strengthening/weakening of conductance due to the application of positive/negative pulses indicates synaptic potentiation and depression, with the device conductance taken as the synaptic weight. Using a series of 25 voltage pulses of 75 ms pulse width and 165 ms time interval, the normalized conductance (synaptic weight) can be changed from 141.6 to 153.1, thereby causing a long-term potentiation(LTP). Similarly, a series of 25 negative voltage pulses were applied, resulting in decrease in the normalized conductance from 150.4 to 136.8 causing long term depression (LTD). These two trends are similar to typical learning and forgetting curves in the biological synapse. From Fig. 4b–e, it can be seen that the overall conductance increases for higher pulse voltages, and the synaptic weight also follows a similar trend.

It is interesting to note that in both SET and RESET cycles, multi-conduction states can be obtained. As shown in the Fig. 4f, the excitatory post synaptic current (EPSC) clearly indicate that the resistance states in NiO nanoparticle based devices can be tuned by choosing appropriate voltage pulse. The measurement of EPSC is described in Figure (S3) (supplementary information). We use a series of voltage pulses of given magnitude and measure the post-synaptic current (PSC). We clearly see that the EPSC grows with the number of pulses. We see an increase in the EPSC signal with increased pulse amplitude. In this way, NiO nanoparticle based device can simulate the operation of a artificial synapse. Further, the device can be operated in multiple resistance states as well. Here we adopt a different pulsing scheme to determine the resistance states. The data shown here are the result of read-write-erase pulse voltage cycles applied to the device and a post synaptic current is monitored. The pulse sequence and post synaptic current measured is shown in detail in Figure (S4) (supplementary information). During each pulse, the device obtains a new resistive state. After switching off the pulse, the device goes back to different HRS state which is monitored as shown in Figure (S4). The resistance state after the voltage pulse is removed can be tuned by applying different amplitudes of voltage pulse. That means, the device can be operated in multiple resistance states. In the Figure (S4) (supplementary information), we plot three such states obtained for different pulse voltages used. This clearly indicates our device can be used in the multi-state operation. If such devices need to be used in the synaptic applications, then the conduction variation should be stable for multiple cycles. As shown in the Figure (S5) (supplementary information), very stable potentiation and depression curves are obtained for this device. The device shows more pronounced potentiation and depression for higher voltages. As indicated by the Figure (S5b), The magnitude of the change of conductance increases linearly with the applied voltage. A similar trend can be seen for the depression curves too. In both cases, we observed that the normalized conductance follows different rates of change. As shown in Figure(S5b)(Supplementary information), the synaptic weight varies with different rates for potentiation and depression. This is a confirmation that our devices can operate at different operating voltages giving rise to stable synaptic functionalities. To understand the rate of change of the synaptic weight during the pulsing, the synaptic signals are fitted using double exponential as

1 $$\begin{aligned} \frac{G}{G_0} = A_{0} + A_{1}\exp \left[ \frac{-(x-x_{0})}{t_{short}}\right] + A_{2}\exp \left[ \frac{-(x-x_{0})}{t_{long}}\right] \end{aligned}$$

where A$_{0}$, A$_{1}$ and A$_{2}$ are constants, x$_{0}$ forms the first pulse of the pulse trains. t$_{short}$ and t$_{long}$ are respective short and long time constants. For various pulse voltages used, the first stage of potentiation and depression curves change faster and was followed by a slower change in both potentiation and depression. This results in two time constants as indicated by t$_{short}$ and t$_{long}$. Individual time constants for both potentiation and depression is shown in Figure S5 (refer the table in Supplementary information). The short and long time constants differ by an order of magnitude for both potentiation and depression. While for potentiation, the ratio remains the same for different pulse voltages, while for depression, the ratio is drastically reduced. The two types of time constants are usually found in the potentiation and depression curves.

**Figure 5:**
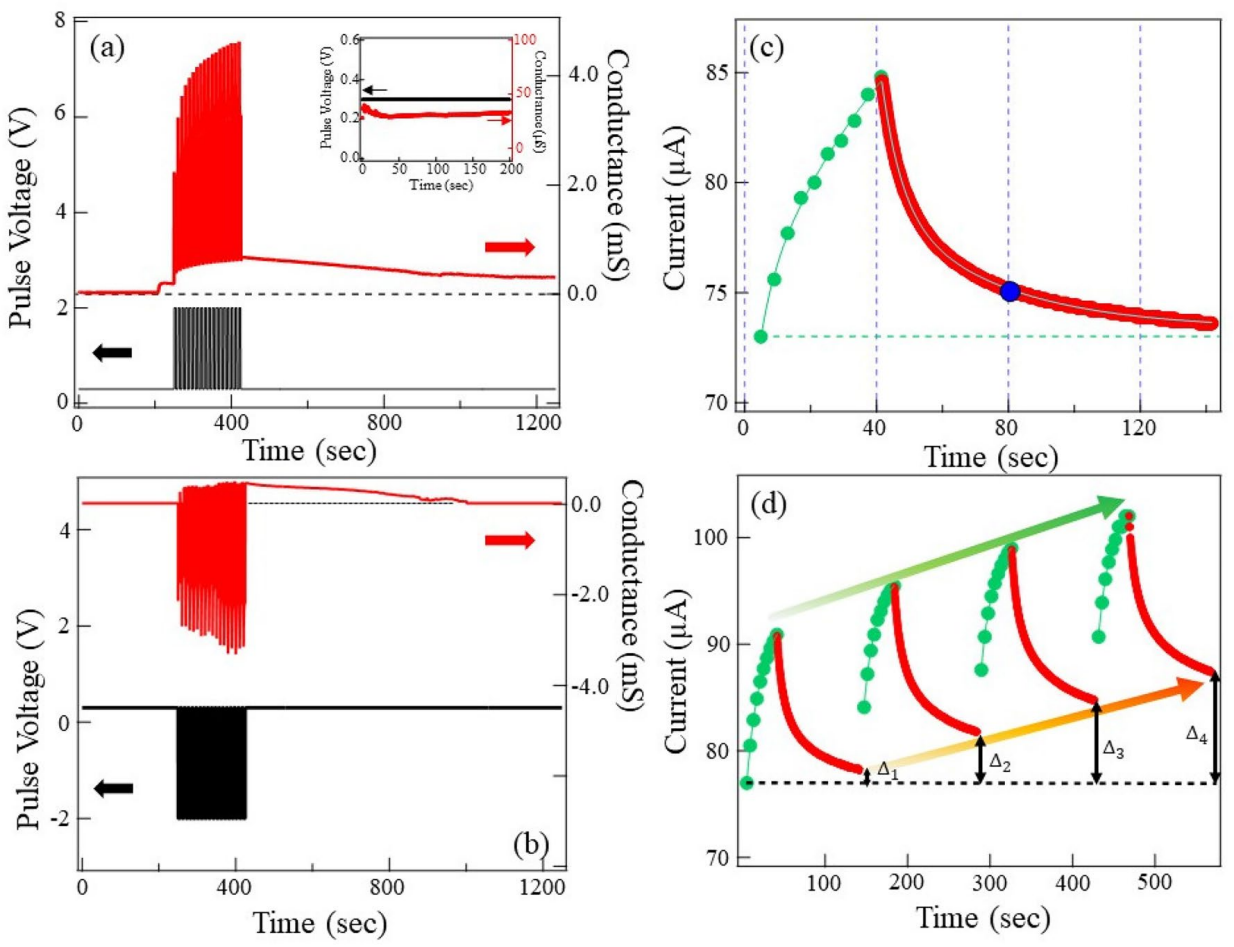
(**a**) Long-term Potentiation (LTP) and Long Term Depression (LTD) are shown by the device. A Series of 25 positive pulses are applied to generate the LTP. At the end of 20 min, about 54% of the post-synaptic current is retained by the device. Similarly, a series of negative voltage pulses show the long-term depression shown by the device. The post-synaptic current reduces pre-synaptic values after 10 min of switching off the voltage pulses. (**c**) Effect of applying a sequence of 10 pulses followed by reading the retained memory with time.

## Discussion

The nonlinearity of the potentiation and depression curves can be tuned by using a non-ideal pulse voltage sequence. As shown in Figure(S6) (supplementary information), the potentiation and depression curves can be modified depending on the pulse sequence used. We tested by taking three types of pulse sequences. In the first type, the voltage pulses with a constant pulse amplitude and pulse width was used. As indicated, we see exponential growth and decay in the potentiation and depression. In the second type, we vary the amplitude of the voltage pulse from 0.3V to ±3V. We can clearly see that for such a non-ideal pulse sequence, the nonlinearity of the potentiation and depression reduces quite drastically. When the pulse-to-pulse time is varied, the potentiation and depression again show growth and decay of synaptic weight depending exponentially. These measurements indicate that the linearity in the potentiation and depression can be achieved using a non-ideal pulse train. If we want to use the our synaptic device in real neuromorphic circuits, a linear synaptic weight update is necessary^54^. But in reality, the synaptic weight update is not linear. One can estimate the deviation from the linear weight update through the nonlinearity factor (NLF) both in the potentiation and depression. We use a similar protocol to calculate the normalized as mentioned previously^54^. The NL value ranges between 0.47 $\sim$ 0.52 for different pulse voltages used in potentiation and depression as shown in Fig. 4g.

The controlled synaptic weight modulation shown by our devices directly indicate the fundamental property of long-term potentiation (LTP) and long-term depression (LTD). The measurement of LTP and LTD is done using a slightly different pulse sequence compared to the normal potentiation and depression curves. The measurement protocol has two parts. The first part is to monitor the synaptic current for a few min, as shown in the inset of Fig. 5a, and then apply a series of 18 voltage pulses and then monitor the post-synaptic current with time. After a series of positive pulses (V$_{P}$ = 2 V), the post-synaptic current is monitored for up to 15 min. At the end of this period, about 54% of post-synaptic current is retained, and we denote this as long-term potentiation (LTP). Similarly, we find that for a series of negative pulses (V$_{P}$ = − 2 V), we see that the post-synaptic current reduces to the pre-synaptic current state within a duration of 10 min, and we term this as long-term depression (LTD). During this interval, the memory is lost from the device. Such a long-term potentiation and long-term depression in a single device have been reported in the literature^55,56^. We used such measurement protocol with fewer pulses to see if long-term potentiation and long-term depression could be seen in the device. For positive pulse voltages, the device shows long-term potentiation (LTP), and for negative voltage pulses, the device shows long-term depression, as shown in Fig. 5b. This clearly indicates that once the device is set for a given memory state, it will be retained for a long time, thus showing long-term memory characteristics. It can be seen that the temporal change in the post synaptic current shows a rapid increase in the current initially followed by a gradual reduction in the post synaptic current. This is highly analogues to the human synaptic behaviour.

It can be found that the temporal evolution law of the photocurrent is a rapid increase at the beginning and then a gradual decrease, which is highly analogous to human synapse behavior.

Memory retention for the long duration is a fundamental characteristics of the sensory organ in humans. It forms an essential feature of as seen in the biological synapses. In particular, the working mechanism of the sensory organ in humans. In the case of the sensory organs of humans, the adjustable synaptic weight is obtained by optimizing the response of the sensory organs to the optical signals. One can mimic similar applications using optimized synaptic weight depending on the number of electrical pulses applied to the device. Such optimization can emulate the learning-forgetting-relearning process. We trained our device with different cycles of pulses and monitored the evolution of synaptic currents. Firstly, we applied 10 voltage pulses, followed by monitoring the synaptic current for a long time as shown in Fig. 5c,d.

Each cycle corresponds to a train of 10 pulses of V$_{P}$ = 2.5 V and V$_{r}$ = 0.3 V, then continuously monitoring the memory for 120 s, as shown in Fig. 5c. The post-synaptic conductance increases in the devices with repeated pulsing, and when the pulsing is switched off, the memory decays, resulting in a forgetting process. This is precisely emulating biological synapses where the partial loss of memory with time after a learning sequence occurs. Our device can retain about 17% of the memory at the end of 40 s indicated by the yellow dot in the decay curve. After 100 s, the device retains about 4% of the memory. However, memory loss with time can be improved by repeated learning or relearning steps. We subjected our device to 4 cycles of learning–forgetting–relearning process, as shown in Fig. 5d. Memory retention is improved with the number of cycles indicated. The post-synaptic conductance increased gradually with the number of cycles. Overall, after the end of 4 cycles, we see that the post-synaptic conductance is an order of magnitude higher than the initial learning step. This is similar to human learning experiences where higher cognitive levels can be achieved by repeated relearning procedures. We observe that during consecutive cycles, the percentage of memory retained remained constant at the end of the 100 s duration, and most importantly, the overall memory increased with the number of pulse cycles. The gradual improvement in the post-synaptic conductance ($\Delta$ I) is indicated by the length of double headed arrow. With the number of cycles memory retention improved as indicated by $\Delta$I$_{1}$ < $\Delta$I$_{2}$ < $\Delta$I$_{3}$ < $\Delta$I$_{4}$. We tested our device for a higher number of the learning-forgetting-relearning cycles as shown in Figure(S5) (supplementary information). it is very clear that the retention of memory can be improved linearly with the number of cycles of learning–forgetting–relearning steps.

To summarize, we have demonstrated a highly efficient NiO nanoparticle-based two-terminal device for artificial synaptic applications. The NiO nanoparticles are synthesized in a simple, facile route, and the two-terminal NiO nanoparticle-based device is fabricated by spin coating on a glass substrate with prefabricated electrodes. The device shows excellent, repeatable analog resistive switching characteristics, which is beneficial for multi-state memory applications. Typical potentiation-depression (learning-forgetting) curves show that the NiO nanoparticle-based devices can be used for memory applications with synaptic functionalities. Synaptic characteristics such as LTP and LTD have been shown by the device. It is also seen that by using an unconventional pulsing method, linearity in potentiation and depression can be achieved. The Learning–forgetting–relearning characteristics have been tested successfully, and the device shows progressive memory retention with the number of learning-forgetting-relearning procedures. This work elucidates an important step towards achieving the nanoparticle-based artificial synapses for neuromorphic applications.

## Methods

### Synthesis of nickel oxide (NiO) nanoparticles

All chemicals and reagents used in this experiment are analytical grade and utilized without further purification. Nickel(II) nitrate hexahydrate ($\ge$97%; Ni(NO$_{3}$)$_{2}$.6H$_{2}$O), Sodium Hydroxide ($\ge$ 98%; NaOH) was purchased from Sigma-Aldrich. Nickel oxide (NiO) nanoparticles were synthesized by the co-precipitation method. In the following reaction, analytical grade (0.6 mM) nickel(II) nitrate hexahydrate (Ni(NO$_{3}$)$_{2}$.6H$_{2}$O) and (1.8mM) sodium hydroxide (NaOH) were completely dissolved in 60 ml deionized water separately. Then sodium hydroxide was added dropwise until (8.0 pH) reached and stirred solution for 3 hours at 600 rpm and increased temperature gradually 80-85$^{\circ }$C. The solution with light green precipitate was centrifuged at 1000 rpm for 30 min and washed several times with ethanol and distilled water, and dried at 120$^{\circ }$C for 12 h. Finally, NiO powder was annealed at 500 $^{\circ }$C for 3 h. The schematic diagram of the synthesis procedure is shown in Fig. 1a. The dried NiO sample was checked for the crystallographic phase using x-ray diffraction spectra. We use CuK$\alpha$ $_{1}$ radiation with a wavelength of 1.5406Å, and the data was collected from 10–90$^\circ$(2$\theta$) with a scanning range of 2$^\circ$/min. Using scanning electron microscopy, we performed morphological studies. The uniform nanoparticle distribution is imaged as shown in Figure (S1) (supplementary information). Large area EDS mapping shows strong Ni K$\alpha$ and O K$\alpha$, indicating a uniform distribution of NiO across a large area. EDS spectrum also displays a strong signal from Ni and O, further corroborating the chemical composition of NiO nanoparticle film (Figure (S1), supplementary information).

### Fabrication of the two terminal devices

We used pre-fabricated, inter-digitated Au electrodes on a glass substrate with 10 $\mu$m separation (Micrux Technologies, Spain). The bare electrodes were ultrasonicated multiple times using isopropanol (IPA) for 30–60 min and dried by keeping them at a slightly elevated temperature. As-prepared NiO nanoparticles were dispersed in ethanol and ultrasonicated for a few hours. The uniformly distributed NiO nanoparticles were drop-casted across the prefabricated electrodes, and the solvent was evaporated by keeping the device at 150$^{\circ }$C for 2–4 hours. A similar step was adopted several times to get uniform nanoparticle film across the Au electrodes. Finally, to minimize the sample-to-sample variation, the device was annealed at 200$^{\circ }$C/10 hours in ambient conditions. The dc-voltage sweeps and the pulsed measurements were done at room temperature using a Keysight B2902B source meter.

## Supplementary Information

Supplementary Information.

## Data Availability

The datasets used and/or analyzed during the current study are available from the corresponding author on reasonable request.
