# Supplementary material for: Bio-inspired artificial synapse for neuromorphic computing based on NiO nanoparticle thin film
Source: Sci Rep. 2023 May 9;13:7481. doi: 10.1038/s41598-023-33752-5 (PMC10169867; doi:10.1038/s41598-023-33752-5)
Supplement: Supplementary file 1 — Supplementary Information. [file 41598_2023_33752_MOESM1_ESM.pdf]

Bio-inspired Artificial synapse for neuromorphic computing based on NiO nanoparticle thin film

[1]Keval Hadiyal, [2]Ramakrishnan Ganesan , [3]A. Rastogiand [3*]R. Thamankar,

[1]*Department of Physics, School of Advanced Sciences, Vellore Institute of Technology, Vellore, Tamilnadu - 632014. India*

[2]*Department of Chemistry, Birla Institute of Technology and Science (BITS), Pilani, Hyderabad Campus, Jawahar Nagar, Kapra Mandal, Medchal District, Hyderabad, Telangana – 500078. India*

[3]*Centre for Functional Materials, Vellore Institute of Technology, Vellore, Tamilnadu - 632014. India*

* Corresponding author
E-mail address: rameshm.thamankar@vit.ac.in

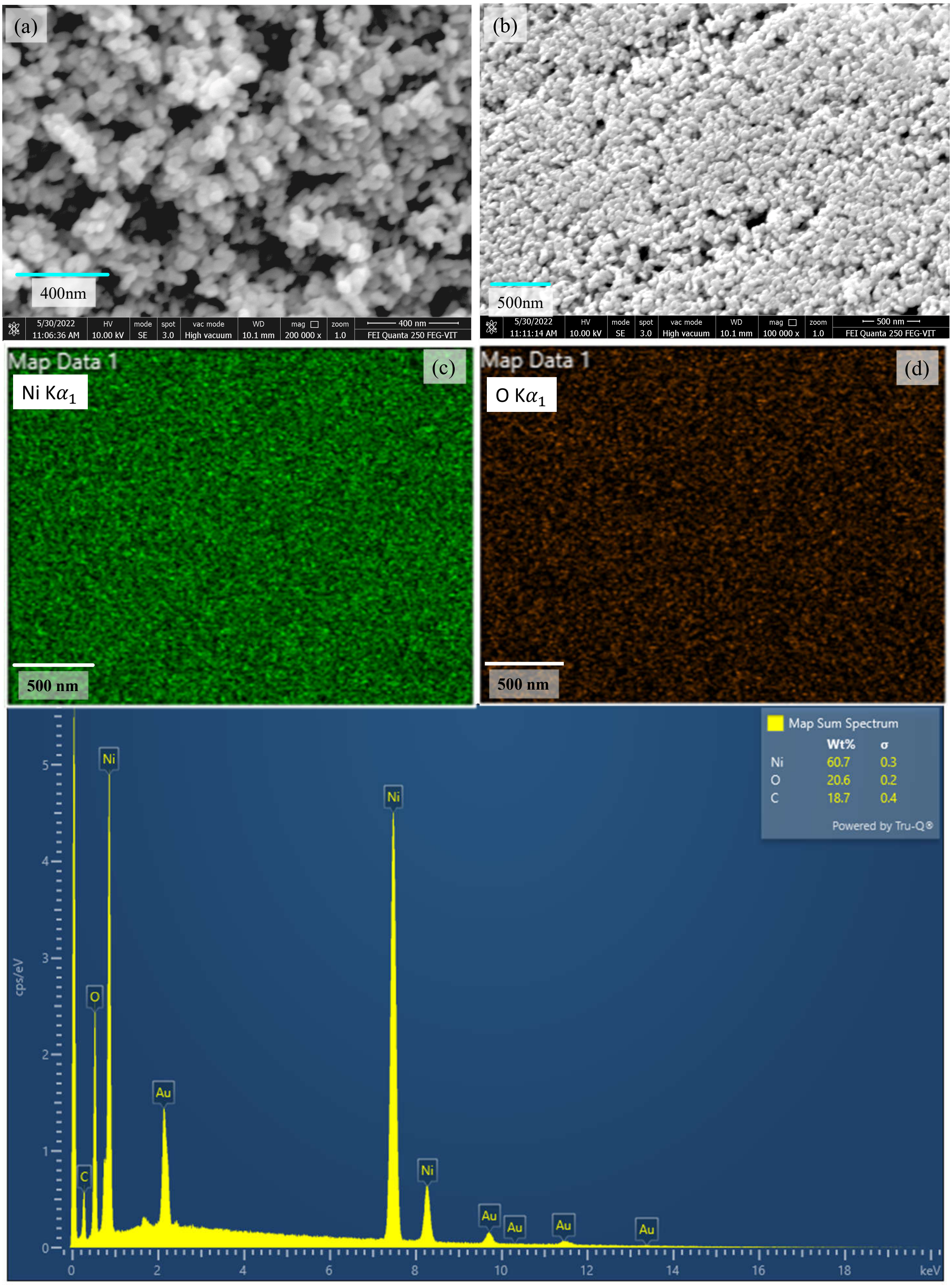

Figure S1 : The Scanning electron Microscopy images of the NiO nanoparticle thin film. (a) and (b) show large area image Depicting uniform distribution of NiO nanoparticles. (c) ED Spectroscopy image for Ni in an area of 3.1 um x 2.4 um and (d) The ED spectroscopy image in the same area depicting the presence of oxygen. (e) Overall E D Spectra shows strong peaks of Ni and O. The average size of the nanoparticle is found to be ~ 45nm.

2θ	FWHM	Crystalline Size D (nm)	Average size (nm)
36.903	0.344	24.345	**23.452**
42.949	0.344	24.816	
62.545	0.394	23.590	
75.071	0.394	25.427	
79.111	0.54	19.081	

Table S2 : Analysis of the XRD peaks (shown in Figure(1)). For the calculation of the crystallite size, all the peaks are considered individually and we used Scherrer formula to calculate the average crystallite size.

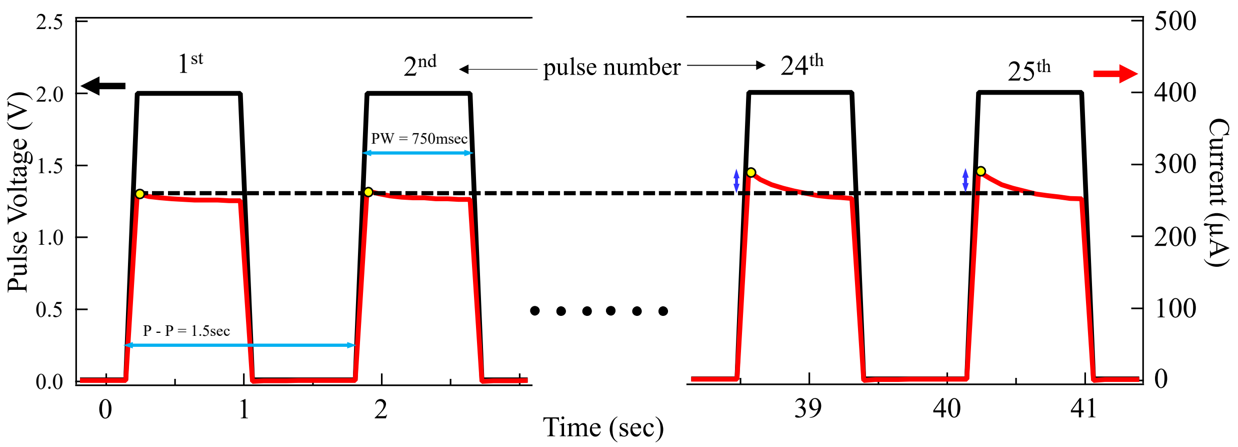

Figure S3 : Evaluation of Excitatory Post Synaptic Current (EPSC) measured using a series of voltage pulses applied to the devices. The black curves indicate the voltage pulses and red curves show the measured EPSC. We applied 25 voltage pulses of different magnitude with pulse width (PW) = 750 msec and pulse – to – pulse time (P-P) of 1.5 sec. The EPSC is measured immediate after the voltage pulse is applied, indicated by the yellow dots on the red curve. The EPSC grows with the number of pulses as indicated with blue double headed arrows. Dashed line is a guide to eye.

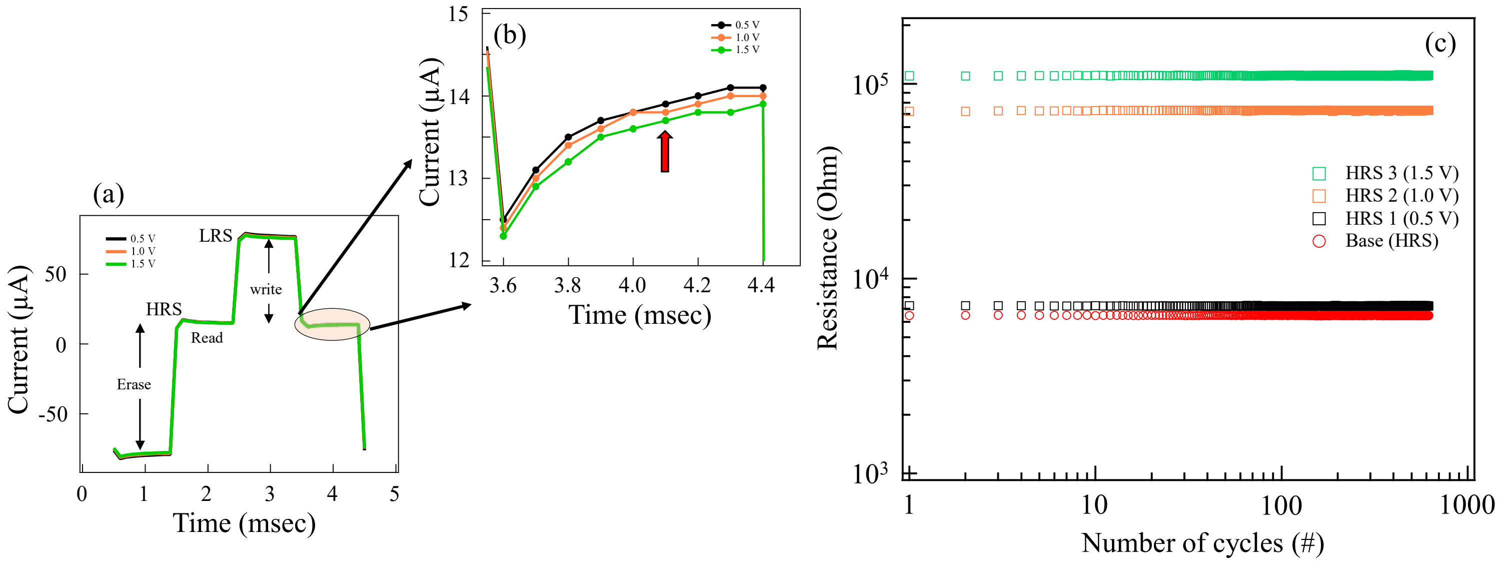

Figure S4: The multi-conduction state operation of the artificial synapse. Read – Write – Erase voltage pulses are applied to the synapse and the post synaptic current is monitored. (a) Single pulse sequence of erase – read – write –red – erase. The erase pulse and write pulses are varied and the post-synaptic current (PSC) is monitored. The device is taken to a low resistance state (LRS) from a previously maintained high-resistance state (HRS). Once the pulse voltage is switched off, the PSC is monitored (b) the HRS state after the pulse voltage switched off. Depending on the pulse voltage, the resistance state can be tuned to achieve multistate operation of the synaptic device. (c) The multiple resistance states obtained for different pulse voltages used in (a). Very stable and consistent resistance states can be obtained for more than 500 cycles of operation. The data points are taken from (b) indicated by the red arrow.

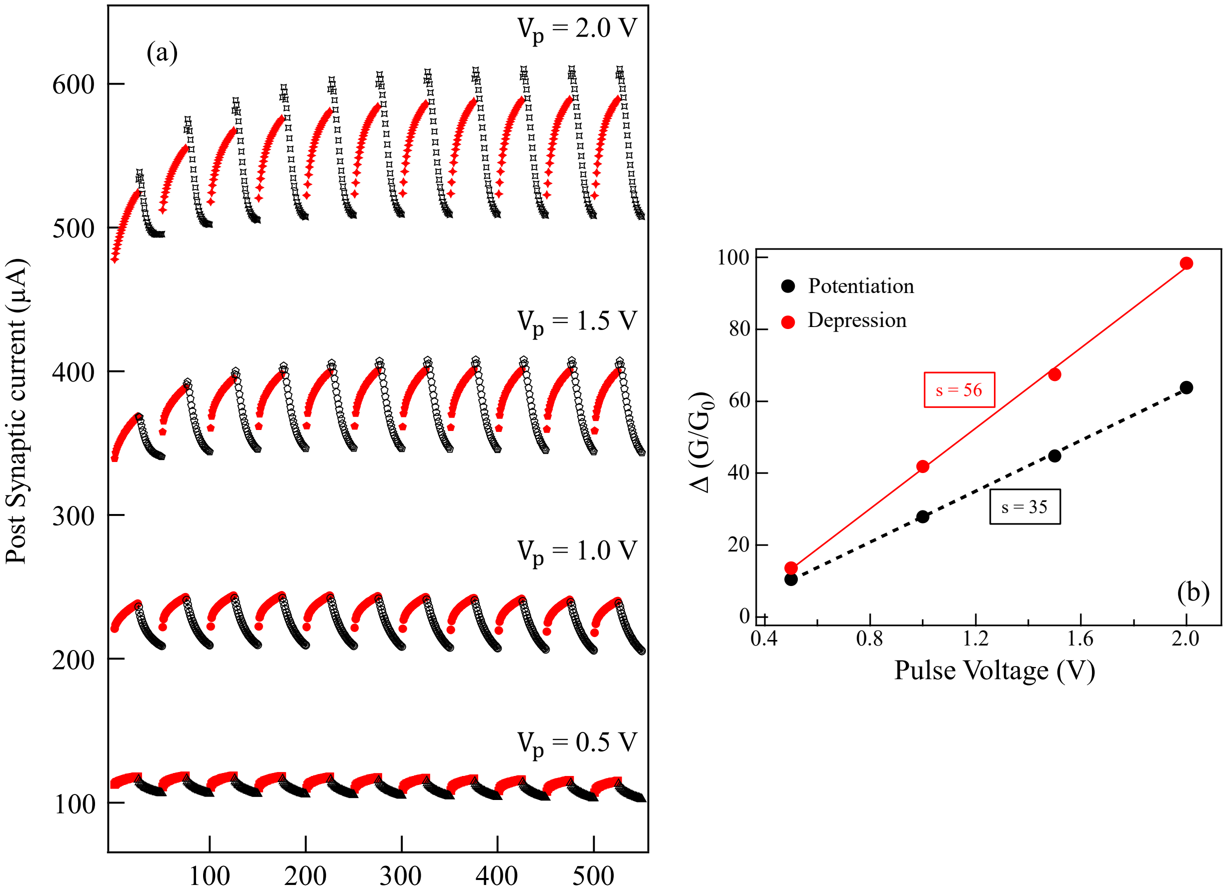

(c) Pulse voltage	Potentiation (t_p)			Depression (t_d)		
	$t_{short,p}$	$t_{long,p}$	$\frac{t_{long,p}}{t_{short,p}}$	$t_{short,d}$	$t_{long,d}$	$\frac{t_{long,d}}{t_{short,d}}$
0.5	0.96	16.38	17.1	1.4542	17.493	12.03
1.0	0.85	18.96	22.31	6.5196	18.021	2.76
1.5	0.88	19.25	21.88	10.796	10.899	1.0086
2.0	2.96	23.11	7.81	6.5	6.3	0.97

Figure S5 : The combined potentiation and depression curves with various values of pulse voltages used. For each pulse voltage, series of 11 cycles are repeated to check the learning – forgetting – relearning process. Overall PSC level increases with the pulse voltage. (b) The relative amplitude of PSC change is more for the potentiation step than the depression step. (c) table showing the time constants for the potentiation and depression. Both potentiation and depression curves are fitted with double exponentials as indicated in the text. Potentiation follows a faster growth ($t_{short,p}$) during initial pulsing and then has a slow variation for subsequent pulsing ($t_{long,p}$). A similar $t_{short,d}$ and $t_{long,d}$ can be associated with the depression curves.

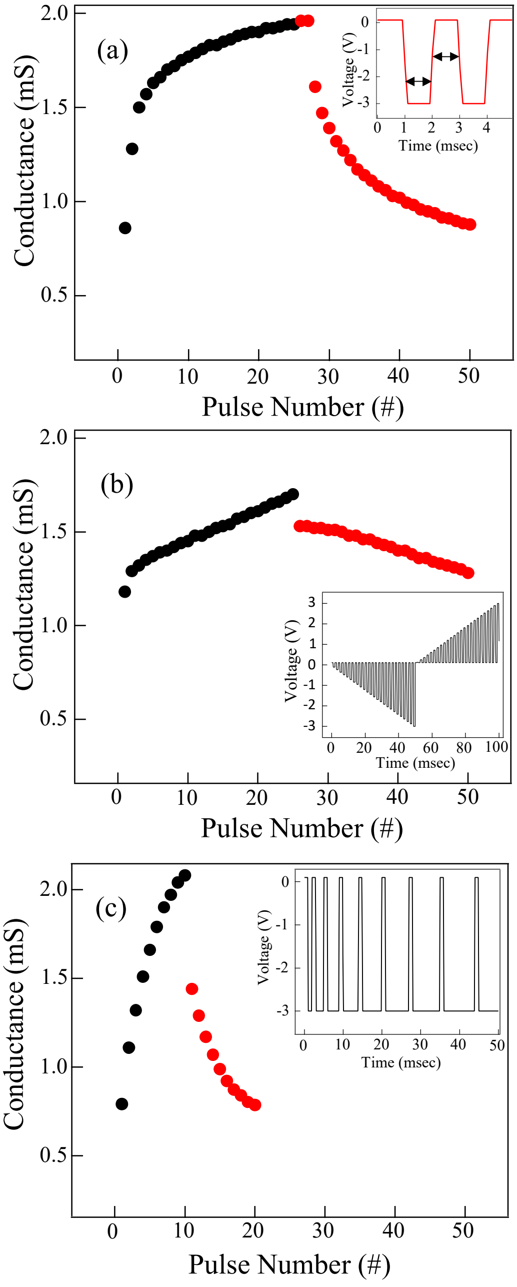

Figure S6 : Application of non-ideal voltage pulses to the device and the neuromorphic performance. (a) The normal constant voltage pulsing results in exponential growth and decay of the post synaptic current. (b) When progressively increasing voltage pulses are used, the potentiation and depression curves tend towards linearity. (c) For a given pulse voltage (-3 V), increasing the pulse width reduces the nonlinearity.

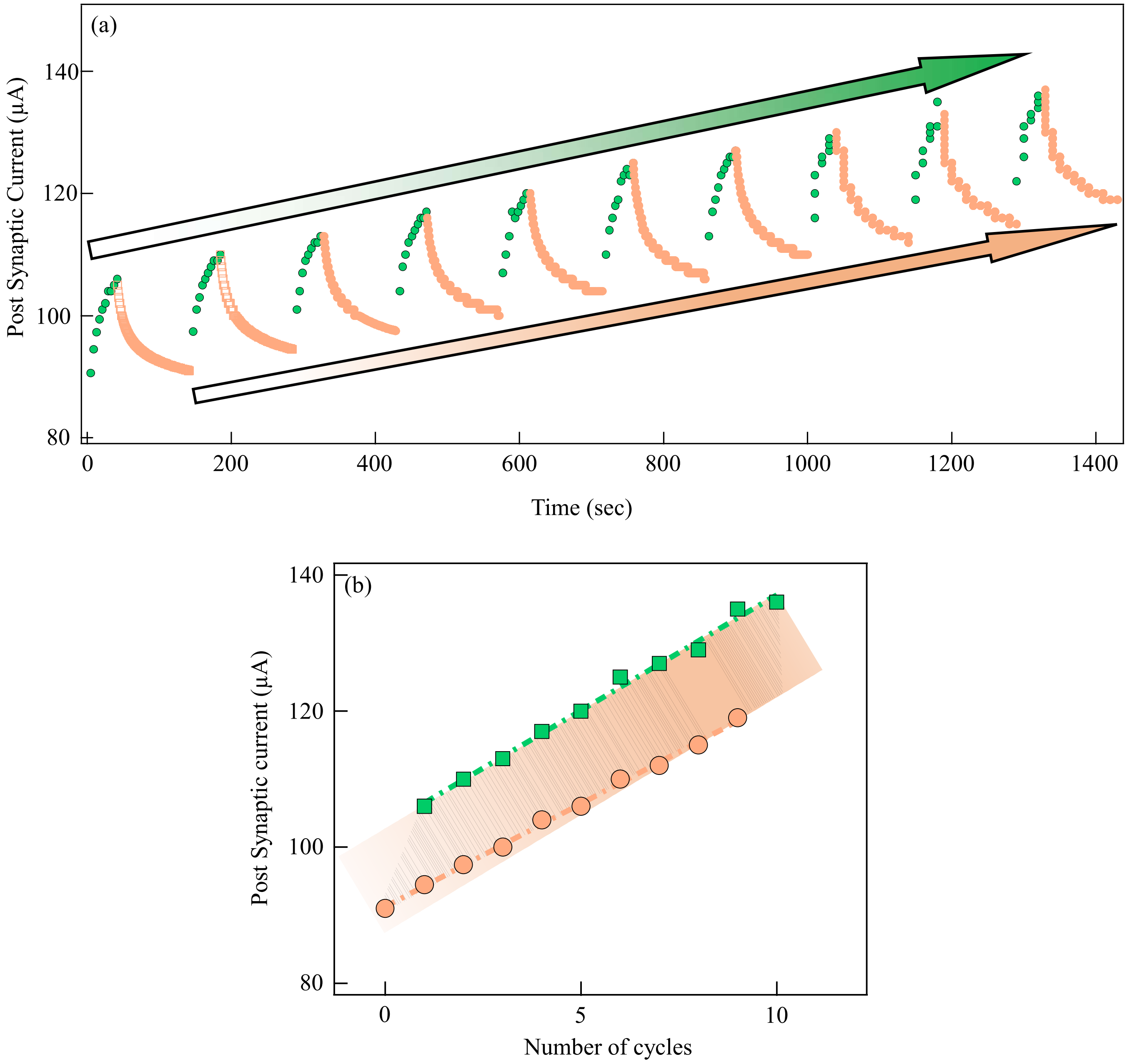

Figure S7 : Emulating the learning – forgetting – relearning curves for NiO nanoparticle based synapse. A series of 10 cycle operation increases the post synaptic current from 90.6 μA to 119 μA. For each Learning curve, 10 pulse () are applied and then the PSC is monitored with time. A linear increase in the PSC can be useful for memory function. (b) The PSC after pulsing (green) and PSC after monitoring for 2 min (orange). Both vary linearly with number of cycles showing an efficient biological synapse using NiO nanoparticles.
